# Can network analysis inform diagnostic and treatment procedures? Evidence and perspectives in eating disorders

**DOI:** 10.1192/j.eurpsy.2025.169

**Published:** 2025-08-26

**Authors:** A. M. Monteleone

**Affiliations:** Department of Psychiatry, University of Campania L. Vanvitelli, Naples, Italy

## Abstract

**Abstract: Introduction:**

The network theory of psychopathology has recently provided a novel view of mental disorders. A growing number of studies employing this methodology has been conducted in eating disorders (ED).

**Objectives:**

Clinical implications of network approach to ED will be discussed spanning diagnosis and classification, comorbidity, prognosis, and treatment.

**Methods:**

Evidence from literature studies and reviews exploring ED psychopathology and treatment through the use of network analysis will be provided. Limitations related to the employment of the network approach will be discussed along with recommendations for future directions.

**Results:**

Overvaluation and concerns about body shape and weight and desire to lose weight are the most central symptoms across patients’ ages and ED diagnoses. Internalizing symptoms, ineffectiveness, and low interoceptive ability play a central role in the psychopathology of ED and promote psychiatric comorbidity. The main issues of network analysis are the selection of network items and the use of cross-sectional data. Prognosis and mechanisms of treatment-induced changes are under-investigated areas.

**Conclusions:**

Network analysis supports a description of ED psychopathology as including eating specific and general psychological symptoms. Treatments designed to improve the most central symptoms and their connections will be essential to explore assumptions of network theory. Longitudinal data from multilevel assessment will allow exploring within person trajectories of psychopathology, observing the dynamic nature of psychopathology and identifying risk and vulnerability factors. This approach will contribute to describe a staging model of EDs, that is essential to improve prognosis formulation and individualize treatment approaches.

**Disclosure of Interest:**

None Declared

